# Vitamin K Insufficiency in the Indian Population: Pilot Observational Epidemiology Study

**DOI:** 10.2196/31941

**Published:** 2022-02-03

**Authors:** Rama Vaidya, Ashok D B Vaidya, Jayesh Sheth, Shashank Jadhav, Umakant Mahale, Dilip Mehta, Janusz Popko, Vladimir Badmaev, Sidney J Stohs

**Affiliations:** 1 Division of Endocrine and Metabolic Disorders Medical and Research Centre Kasturba Health Society Mumbai, Maharashtra India; 2 Medical Research Centre Kasturba Health Society Mumbai India; 3 The Foundation for Research in Genetics and Endocrinology Ahmedabad, Gujarat India; 4 Synergia Life Sciences Pvt Ltd Mumbai, Maharashtra India; 5 Medical University of Bialystok Bialystok Poland; 6 Creighton University Medical Center Frisco, TX United States

**Keywords:** phylloquinone, menaquinone-7, vitamin K1, vitamin K2, insufficiency, deficiency, Indian population, diabetes, healthy people

## Abstract

**Background:**

The fat-soluble K vitamins K1 and K2 play an essential role in the blood coagulation cascade and are made available predominantly through selective dietary intakes. They are less known for their nonessential roles in a family of vitamin K–dependent proteins that promote various functions of organs and systems in the body. A lack of vitamin K can characterize vitamin and nutritional element insufficiency, which is different from a clinically apparent vitamin deficiency.

**Objective:**

This epidemiological study evaluated the nutritional status of vitamin K in a sample of the Indian population and vitamin K content in staple Indian foods.

**Methods:**

Serum levels of vitamin K1 and vitamin K2 in the form of menaquinone-7 (MK-7) were assessed via high-performance liquid chromatography coupled with fluorescence detection in 209 patients with type 2 diabetes, 50 healthy volunteers, and common staple foods in India.

**Results:**

After comparing populations with high and low serum vitamin K levels from various geographical regions, our results indicated that the sample of healthy Indian individuals and the sample of Indian patients with type 2 diabetes had low (insufficient) levels of vitamin K2 (MK-7; range 0.3-0.4 ng/mL). No significant differences existed in vitamin K1–related and MK-7–related values between healthy male and female subjects, between male and female subjects with diabetes, and between the healthy sample and the sample of patients with diabetes. The staple, commonly consumed Indian foods that were tested in this study had undetectable levels of vitamin K2, while levels of vitamin K1 varied widely (range 0-37 µg/100 g).

**Conclusions:**

Based on our sample’s low serum levels of vitamin K2 (MK-7) as well as the low levels of vitamin K2 in their typical diet, we propose that the general Indian population could benefit from the consumption of vitamin K2 in the form of MK-7 supplements.

**Trial Registration:**

Clinical Trials Registry - India CTRI/2019/05/014246; http://ctri.nic.in/Clinicaltrials/showallp.php?mid1=21660&EncHid=&userName=014246; Clinical Trials Registry - India CTRI/2019/03/018278; http://ctri.nic.in/Clinicaltrials/showallp.php?mid1=32349&EncHid=&userName=018278

## Introduction

Clinically apparent nutritional deficiency diseases, such as scurvy (vitamin C deficiency), rickets (vitamin D deficiency), beriberi (vitamin B1 deficiency), and pellagra (vitamin B3 deficiency), differ from nutritional insufficiency—a relatively new finding. The effects of vitamin insufficiency with regard to a number of vitamins are well known. However, the roles of vitamin K, which are exemplified by the insufficiency of this vitamin, are much less well known [1,2].

Recently, it has been found that the mechanism of nutritional insufficiency can be explained by a triage (sort and select) mechanism, and nutritional insufficiency can be exemplified by the nutritional insufficiency of vitamin K [2]. The term *triage* is used on the battlefield to prioritize treatments for the survival of the wounded. In a similar way, the human body prioritizes nutrient use (ie, micronutrients, nutrients, and vitamins) for immediate needs (eg, survival) over nutrient use for long-term needs (eg, sustaining life) by borrowing nutrients from less critical depots in the body, always securing the emergency requirements.

In healthy people who consume a varied diet, the clinical deficiency of vitamin K is rarely encountered because the body prioritizes available vitamins for use with essential, life-sustaining, vitamin K–dependent proteins (eg, the carboxylation of such proteins and the activation of vitamin K–dependent blood coagulation proteins, such as coagulation factors II [thrombin], VII, IX, and X). However, vitamin K may be insufficient for at least 18 vitamin K–dependent, nonessential, calcium-dependent proteins that are responsible for healthy cardiovascular, immune, skeletal, and neuromuscular systems [1,3].

Due to the recent discovery of its many biological functions, vitamin K is known as a “multitasking” vitamin. In the long term, the insufficient status of this multitasking vitamin may prevent vitamin K–dependent nonessential proteins from functioning optimally and result in the development of chronic degenerative conditions, such as osteoporosis, cardiovascular disease, metabolic conditions (eg, diabetes), and neurodegenerative conditions, that ultimately diminish quality of life and shorten the life span [1].

Due to the growing awareness of the importance of vitamin K nutritional status, the objectives of this epidemiological study were to evaluate serum levels of vitamin K in healthy men and women and patients with diabetes selected from the Indian population and to assess the average vitamin K content in the indigenous diet of India. This study evaluated 2 fat-soluble vitamins—vitamin K1 (phylloquinone), which is commonly found in a diet of green vegetables and plant margarine, and vitamin K2 (menaquinone; specifically, menaquinone-7 [MK-7]), which is derived mainly from meat; liver; butter; egg yolks; fermented foods (eg, cheese and curd); and indigenous Indian dietary products, including dosa, dhokla, and yogurt.

Besides food sources, vitamin K2 is also synthesized in the human gut microbiome predominantly by *Bacteroides* and *Veillonella* bacteria [4]. However, gut bacteria that generate menaquinones reside mostly in the large intestine. As such, gut bacteria–derived menaquinones are less bioavailable, since the absorption of menaquinones occurs predominantly in the small intestine. Therefore, serum levels of menaquinones depend largely on dietary sources [4].

## Methods

### Subject Selection

The Department of Medicine at Kokan Hospital in Mumbai, India, selected a sample of the native population based on the admission criteria for this study, which are provided in Textbox 1.

The Inter System BioMedica Ethics Committee—an independent ethics committee—approved the protocol of this study and registered this study with Clinical Trials Registry - India (trial CTRI/2019/05/014246 for the healthy population and trial CTRI/2019/03/018278 for the population with diabetes). The trial registration information can be found on the Clinical Trials Registry - India website and the World Health Organization portal. The Department of Medicine at Kokan Hospital in Mumbai, India, approved the study protocol according to the International Conference on Harmonization of Good Clinical Practice Guidelines [5].

The sample of patients with diabetes (n=209) was randomly selected from patients who visited the hospital diabetes clinic for treatment, and the healthy subjects (n=50) were screened for their health status and criteria of inclusion upon responding to the advertisement for study participants. Those who met the inclusion criteria were selected for this study. The data obtained were recorded in ethics committee–approved case record forms. The participants agreed with and signed the informed consent form prior to their admission to the study groups, and blood samples were collected following a fasting period of at least 8 hours and after the consumption of a standard meal.

**Inclusion and exclusion criteria for the healthy population and the population with diabetes.**

**Inclusion criteria**

- Healthy population
  Healthy males and females aged 28 to 45 years
  Having a BMI of 18.5-24.9 kg/m^2^
- Population with diabetes
  Males and females aged ≥25 years
  A duration of diabetes of ≥6 months from the date of diagnosis
  Fasting plasma glucose levels of ≥126.0 mg/dL

**Exclusion criteria**

- Healthy population
  People with any systemic illness
  People who are on corticosteroids and oral contraceptives
  People on antibiotics within the last week
  People with a seropositive status
  Pregnant and lactating women
  Participation in clinical trials evaluating investigational pharmaceuticals or biologics within 3 months of admission to our study
  Participation in clinical trials evaluating devices within 30 days of admission to our study
  People who are on coumarin analogues or quinine hydrochloride
  People who have a history of smoking, alcohol abuse, or substance abuse within the last week
- Population with diabetes
  Subjects with type 1 diabetes
  Lactating and pregnant mothers
  Participants with concomitant chronic illness

### Analytical Methods

The serum levels of vitamin K1 and vitamin K2 in the form of MK-7 were assessed via high-performance liquid chromatography (HPLC) coupled with fluorescence detection. The vitamin K1 and vitamin K2 (MK-7) content in food was evaluated with the same HPLC analytical method. A reverse-phase HPLC method [6-8] involving postcolumn derivatization and fluorescent detection was used, with some modifications. This method was validated according to the International Conference on Harmonization guidelines [5]. Our laboratory works with the UK Vitamin K External Quality Assurance Scheme (KEQAS) to ensure quality control.

Vitamin K1 and vitamin K2 (MK-7) analytical standards, a vitamin K2-6 internal standard, and zinc dust (<10µM) for the postcolumn reduction of vitamin K were purchased from Sigma Chemical Co. All of the other chemicals used were analytical reagent grade, and all solvents for HPLC were HPLC grade.

An HPLC system (Shimadzu Corporation) was used. This system was equipped with a degasser (DGU-20A5R; Shimadzu Corporation), pump (LC-20AD; Shimadzu Corporation), auto-sampler (SIL-20AC HT; Shimadzu Corporation), column oven (CTO-10 AS; Shimadzu Corporation), and fluorescence detector (RF 20A; Shimadzu Corporation). A reverse-phase C18 column (Kinetex C18 [Phenomenex Inc]; size: 100×4.6 mm; volume: 2.6 µL; angle: 100°) was used, and the column oven was adjusted to 25 °C. A postcolumn (30×4 mm) assembly was inserted between the analytical column, which was packed with zinc dust for the postcolumn reduction of vitamin K, and the fluorescence detector. The mobile phase contained 2 mL of zinc solution (0.136% zinc chloride, 0.04% sodium acetate, and 30 µL of acetic acid in 75% methanol) in 2 L of methanol and was filtered through 0.45-µm filter paper. Injected samples or standards were eluted at an isocratic flow rate of 1.2 mL per minute over a 12-minute run time. Eluted peaks were monitored with the fluorescent detector, which was set at an excitation wavelength and emission wavelength of 248 nm and 430 nm, respectively.

Six-point calibration curves of vitamin K were created by spiking plasma samples with 0.16- to 20-ng/mL concentrations of the vitamins. All samples were extracted as follows. To each plasma sample, 500 µL of the internal standard (20-ng/mL vitamin K2-6 in methanol) was added, followed by 3.5 mL of ethanol and 0.5 mL of sodium chloride (0.9% in water). Each mixture was vortexed for 2 minutes, and 10 mL of hexane was added. Afterward, the tubes were again vortexed. The hexane layers were separated via centrifugation, collected in separate tubes, and evaporated under nitrogen. The residues were dissolved in 100 µL of methanol, and 50 µL of this solution was injected into the column. For all food samples, about 1000 mg of each test sample was placed in a 100-mL volumetric flask, to which 10 mL of tetrahydrofuran, 1 mL of the internal standard, and 70 mL of ethanol were added. The contents of these flasks were sonicated for 1 hour and diluted to volume with ethanol. Afterward, the samples were filtered, and aliquots of the filtrate were injected into the column.

### Statistical Methods

All data in this study were analyzed by using the 2-tailed unpaired *t* test method. This method was used to determine the statistical significance between 2 sets of data (set at *P*<.05). In addition, the data were analyzed via an analysis of variance to determine their statistical significance between and across all groups. All statistical analyses were conducted by using the built-in functions of Microsoft Excel. Statistical analyses were conducted between healthy male and female subjects, between male and female subjects with diabetes, and between the healthy sample and the sample of patients with diabetes.

### Data Sharing

The data described in this paper, the code book, and the analytic code can be made available upon request.

## Results

### Statistical Analysis

Based on a power calculation and the use of a power calculation table [9,10], a sample size of 25 for each group had 90% power to detect a 0.38 difference between the means of the two groups at a significance level (*α*) of <.05 via a 2-tailed *t* test. A sample size of 100 subjects for each group had 90% power to detect a 0.18 difference between the means of the two groups at a significance level of <.05.

### Demographic and Clinical Data

Baseline demographic and clinical data are presented in Table 1 for male and female subjects in the group of subjects with diabetes and the healthy volunteer comparison group. Table 1 provides the basic demographic details for all subjects with respect to age, gender, and BMI as well as baseline values for systolic and diastolic blood pressure, fasting blood sugar level, postprandial blood sugar level, and glycosylated hemoglobin (HbA_1c_) level in the two study groups based on gender.

Statistically significant differences (*P*<.05) existed between the group of subjects with diabetes and the healthy volunteers with respect to blood sugar levels and HbA_1c_ levels, as would be expected (Table 1). There were no significant differences between male and female subjects within the two groups with respect to these markers. Blood pressure values and BMIs tended to be higher among the subjects with diabetes.

**Table 1:** Baseline demographic and clinical data of the study population.

Characteristic	Study 1 (volunteer patients with type 2 diabetes mellitus: n=209)		Study 2 (healthy volunteers: n=50)	
	Males	Females	Males	Females
Subjects, n	100	109	25	25
Age (years), mean (SD)	50.3 (13.1)	48.6 (14.0)	41.6 (12.8)	36.0 (5.4)
BMI (kg/m^2^), mean (SD)	25.4 (4.2)	26.9 (5.4)	23.8 (2.3)	22.7 (1.9)
HbA_1c_^a^ level (%), mean (SD)	7.6 (1.7)^b^	7.2 (1.7)^b^	4.9 (0.4)^b^	4.7 (0.4)^b^
Systolic blood pressure (mm Hg), mean (SD)	128.6 (15.6)	126.8 (16.7)	114.6 (10.4)	122.6 (12.9)
Diastolic blood pressure (mm Hg), mean (SD)	89.8 (21.5)	86.4 (22.9)	80.0 (4.8)	80.8 (6.8)
Fasting blood sugar level (mg/dL), mean (SD)	123.2 (36.9)^b^	117.2 (47.3)^b^	82.1 (7.7)^b^	82.5 (8.2)^b^
Postprandial blood sugar level (mg/dL), mean (SD)	193.6 (66.0)^b^	179.9 (83.1)^b^	102.5 (7.1)^b^	104.9 (7.0)^b^

^a^HbA_1c_: glycosylated hemoglobin.

^b^Values are significant at the *P*<.05 level.

### Vitamin K Blood Levels

The levels of vitamin K2 (MK-7) and phylloquinone (vitamin K1) in the healthy population and the population with type 2 diabetes mellitus are presented in Table 2. The differences in the levels of vitamin K1 and MK-7 between male and female subjects with diabetes and between healthy male and female subjects were not statistically significant (*P*<.05) based on the analysis of variance . There was a trend toward higher MK-7 levels in both male and female subjects with diabetes. The vitamin K1 serum levels in the studied healthy population and the population with diabetes were within the normal range, based on the physiological levels of vitamin K1 in their serum samples. The determination of a low vitamin K2 (MK-7) level (range: 0.116-1.056 ng/mL) was based on limited epidemiological data [11,12] from an adult population [13]. However, the serum levels of vitamin K2 in the form of MK-7 in both the healthy sample and the sample of patients with diabetes were determined to be in the low range.

**Table 2:** Levels of menaquinone-7 (MK-7; vitamin K2) and phylloquinone (vitamin K1) in the healthy population and the population with type 2 diabetes mellitus.

Characteristic	Patients with type 2 diabetes mellitus		Healthy volunteers	
	Males	Females	Males	Females
Subjects, n	100	109	25	25
MK-7 level (ng/mL), mean (SD)	0.41 (0.37)	0.42 (0.49)	0.31 (0.23)	0.39 (0.20)
Phylloquinone (vitamin K1) level (ng/mL), mean (SD)	0.55 (0.50)	0.53 (0.46)	0.70 (0.65)	0.48 (0.39)

### Vitamin K Levels in Selected Foods

Table 3 provides data regarding the analysis of vitamin K2 (MK-7) and vitamin K1 content (µg/100 g or µg/100 mL) in staple foods of India. As can be seen in Table 3, vitamin K2 (MK-7) was absent from or below the limits of detection in all studied foods, whereas the vitamin K1 content varied widely. Therefore, the dietary intake of these foods would result in wide variations in vitamin K1 serum levels.

It should be noted that the analytical laboratory involved in this study works with the UK KEQAS to ensure quality control. To ensure the accuracy of the analytical procedures for vitamin K, on 6 occasions, the laboratory received 2 serum samples and 1 standard vitamin K sample from KEQAS for the analysis of vitamin K via the HPLC method. On all 6 occasions, the analytical method that was used yielded satisfactory results, and a “Green” certification was awarded to the laboratory. The target for results was a 20% deviation from the all-laboratory trimmed mean representing the target concentrations of the unknown samples that were provided by KEQAS.

**Table 3:** Staple foods in India that were evaluated for menaquinone-7 (MK-7; vitamin K2) and phylloquinone (vitamin K1) content.

Food item^a^	Description	MK-7 content, mean (SD)	Phylloquinone (vitamin K1) content, mean (SD)
Dhokla	Fermented batter derived from rice and split chickpeas	—^b^	2.76 (0.17)^c^
Naan	Bread leavened with yeast or with a bread starter (flatbread)	—	—
Jalebi	A sweet snack made with deep-fried wheat flour	—	—
Idli	A savory cake made of fermented lentils and rice	—	1.67 (2.13)^d^
Handvo	A cake based on gram flour with vegetables and peanuts	—	37.14 (3.02)^d^
Yogurt	A dairy product made by coagulating milk with any culinary acid (eg, lemon juice)	—	—
Cheese	Processed cheddar cheese	—	4.71 (0.68)^d^
Buttermilk	A fermented dairy drink made by churning the butter out of cultured cream	—	0.51 (0.05)^c^
Butter	Made of pure milk fat	—	4.60 (0.82)^d^
Milk	Cow milk	—	0.97 (0.19)^c^
Charoli	Almond-flavored seeds of a bush (*Buchanania ianzan*)	—	2.85 (0.40)^d^

^a^Four samples from each food item were analyzed.

^b^Not available (not detected).

^c^µg/100 mL

^d^µg/100 g

## Discussion

### Vitamin K Blood Levels in India

The primary outcome of this study was the determination that vitamin K2 insufficiency occurred in both a healthy cohort and a cohort of people with diabetes from India. A secondary outcome was that vitamin K2 was not restricted to individuals with a disease such as diabetes; it was also detected in a group of apparently healthy individuals.

Although the typical nutritional intake in India may provide sufficient vitamin K for supporting the life-sustaining blood coagulation cascade, the vitamin K content of staple Indian foods and the intake of vitamin K from these foods may be insufficient for this vitamin to fulfill its multitasking role in preventing disease and sustaining health in a growing number of people with vitamin K–dependent health conditions. As such, the general consumption of vitamin K2 in the form of MK-7 supplements may be justified.

### Vitamin K Blood Levels Throughout the World

Studies have indicated that serum levels of vitamin K vary throughout the world and are largely dependent upon dietary intake [11]. For example, the serum levels of vitamin K2 (MK-7) in Japan have been reported to differ by region, varying from 5.26 (SD 6.13) ng/mL among women in eastern Japan (eg, Kanto region, Tokyo) to 1.22 (SD 1.85) ng/mL among women in western Japan (eg, Kasai region, Hiroshima) [11]. Further, the mean vitamin K2 (MK-7) level in British women has been reported to be 0.37 (SD 0.20) ng/mL [11].

The recommendations for the daily intake of vitamin K have been inconsistent, in part due to the unquantified vitamin K contribution of intestinal bacteria. For example, the recommended levels of vitamin K that may be adequate for blood clotting are insufficient for other functions [12]. A daily intake of 1 µg of vitamin K per 1 kg of body weight may be adequate for blood clotting [12]. In a Western diet, the intake of vitamin K is estimated to range from 60 µg to 200 µg per day, of which phylloquinone (vitamin K1) constitutes about 90% and menaquinones (vitamin K2) constitute about 10% [14].

Findings for vitamin K2 (MK-7) serum levels indicate wide variations in dietary vitamin K2 content resulting from different dietary staples with varying vitamin K2 contents [11]. For example, fermented beans known as *natto* are consumed regularly at breakfast in eastern Japan, and approximately 1000 µg of MK-7 is provided per 100 g of natto. However, according to an epidemiological study, consuming natto is an infrequent dietary practice in the western part of Japan [11]. This provides a possible explanation for the occurrence of higher vitamin K2 (MK-7) serum levels in women from eastern Japan than those in women from western Japan and may explain the lower incidence of cardiovascular and skeletal morbidity in the former group [11].

### Vitamin K and COVID-19

One of the recently discovered health conditions that has been linked with vitamin K insufficiency is SARS-CoV-2 infection, which results in the viral disease known as *COVID-19* [13]. The scientists behind this epidemiological finding proposed that insufficient serum levels of vitamin K could result in the enhancement of the inflammatory response associated with COVID-19, which contributes to multi-organ failure in patients with COVID-19. This hypothesis is supported by another epidemiological study on vitamin K, in which patients with COVID-19 exhibited reduced vitamin K status and had poor prognoses [11]. Further studies are needed to explore and affirm the possible role of vitamin K in susceptibility to and recovery from COVID-19 as well as other viral diseases, such as influenza.

### Conclusions

The results showed that the serum levels of vitamin K2 in the form of MK-7 among both the healthy sample and the sample with diabetes from the population that was studied were in the low range. As such, the results of this study suggest but do not prove that vitamin K insufficiency may be a common occurrence. The results provide a basis and template for future studies and may also serve as a guide for health care practitioners when it comes to vitamin K2 supplementation. Based on the results of this study, which indicated low serum levels of vitamin K2 (MK-7) as well as low vitamin K2 levels in the typical Indian diet, we propose that the general Indian population could benefit from the consumption of vitamin K2 in the form of MK-7 supplements. Patients with diabetes, elevated blood pressure (a harbinger of cardiovascular disease), and compromised immune systems may especially benefit from MK-7 supplementation. Although this study was conducted in India, the results may be extrapolated to other countries worldwide.

## Abbreviations

HbA_1c_: glycosylated hemoglobin
HPLC: high-performance liquid chromatography
KEQAS: Vitamin K External Quality Assurance Scheme
MK-7: menaquinone-7
